# Caffeic acid derivative MPMCA suppresses osteoclastogenesis and facilitates osteoclast apoptosis: implications for the treatment of bone loss disorders

**DOI:** 10.18632/aging.206067

**Published:** 2024-08-26

**Authors:** Le Huynh Hoai Thuong, Chin-Jung Hsu, Hsien-Te Chen, Yueh-Hsiung Kuo, Chih-Hsin Tang

**Affiliations:** 1 Graduate Institute of Biomedical Sciences, China Medical University, Taichung, Taiwan; 2 School of Chinese Medicine, China Medical University, Taichung, Taiwan; 3 Department of Orthopedic Surgery, China Medical University Hospital, Taichung, Taiwan; 4 Department of Sports Medicine, China Medical University, Taichung, Taiwan; 5 Department of Chinese Pharmaceutical Sciences and Chinese Medicine Resources, China Medical University, Taichung, Taiwan; 6 Chinese Medicine Research Center, China Medical University, Taichung, Taiwan; 7 Department of Pharmacology, School of Medicine, China Medical University, Taichung, Taiwan; 8 Department of Medical Laboratory Science and Biotechnology, College of Medical and Health Science, Asia University, Taichung, Taiwan; 9 Department of Medical Research, China Medical University Hsinchu Hospital, Hsinchu, Taiwan

**Keywords:** MPMCA, osteoclastogenesis, apoptosis, MAPK, NF-κB

## Abstract

Osteoclast activity plays a crucial role in the pathological mechanisms of osteoporosis and bone remodeling. The treatment of these disorders involves the use of pharmacological medicines that work by inhibiting the activity of osteoclasts. Nevertheless, the prevalent and infrequent negative consequences of current antiresorptive and bone anabolic treatments pose significant drawbacks, hence restricting their prolonged administration in patients, particularly those who are elderly and/or suffer from many medical conditions. We are currently in the process of creating a new molecule called N-(4-methoxyphen) methyl caffeamide (MPMCA), which is a derivative of caffeic acid. This compound has shown potential in preventing the production of osteoclasts and causing existing osteoclasts to undergo cell apoptosis. Our investigation discovered that MPMCA hinders osteoclast function via suppressing the MAPK pathways. The expectation is that the findings of this study will stimulate the advancement of a novel approach to treating anti-resorption.

## INTRODUCTION

Bone turnover is essential for maintaining calcium homeostasis and repairing bone defects resulting from microfractures [1]. The process involves bone resorption occurring within approximately 10 days, followed by bone formation taking about three months. Approximately 20% of the skeleton undergoes remodeling every year. Bone turnover markers are categorized as bone formation and resorption markers [2]. Examples of bone resorption markers released during remodeling include tartrate-resistant acid phosphatase (TRAP), bone sialoprotein (BSP), and cathepsin K (CTSK). Bone formation markers include byproducts of collagen formation (propeptides of type 1 collagen C-terminal (P1CP)), osteoblast enzymes (e.g., alkaline phosphatase-ALP), and matrix proteins (e.g., osteocalcin (OC)). During bone remodeling, osteoclasts (bone-resorbing cells) and osteoblasts (bone-forming cells) play crucial functions. Osteoclast-mediated bone resorption is vital in the early stages, while osteoblasts are crucial during bone remodeling and synthesis by formatting and mineralizing new bone [3]. Bone remodeling occurs when osteoclasts are activated, leading to bone resorption preceding bone formation [4]. Defective genes during osteoclast formation can disrupt bone homeostasis [5], potentially leading to osteoporosis [6].

Osteoporosis, a significant public health concern, results in bone fractures [7]. The continuous modeling and remodeling processes manage bone strength and firmness [8]. Osteoporosis is featured by bone loss, the destruction of bone microstructure, and fragility leading to fractures [9]. Despite the constant renewal of bone [10], osteoclastogenesis and osteoclastic bone resorption play pivotal roles in osteoporosis [11], followed by osteoblastic activity [4, 5]. Thus, osteoclasts and osteoblasts play pivotal roles in bone remodelling [12]. Osteoclasts participate in bone disorders and pathogenesis [13]. Osteoclasts originate in hematopoietic tissue or as a result of bone marrow-osteoclast differentiation involving monocytes or macrophages subjected to macrophage colony-stimulating factor (M-CSF) or receptor activator of nuclear factor kappa-Β ligand (RANKL) [13].

Various drugs with different mechanisms effectively inhibit osteoclast function in osteoporosis and osteolytic bone metastasis, but they are linked with potentially different side effects [5]. Traditional Chinese medicine (TCM) compound, caffeic acid (CA), found abundantly in coffee, fruits, vegetables, essential oils, and tea [14], has various beneficial biological effects, including antiviral activities and the ability to lower oxidative stress [15, 16]. CA also inhibits reactive oxygen species (ROS) and osteoclast differentiation [14]. In addition, CA has the potential to regulate the bone remodeling process by inhibiting osteoclastogenesis and bone resorption [14]. CA and its major derivatives, such as caffeic acid phenethyl ester (CAPE) and caffeic acid 3,4-dihydroxy-phenethyl ester (CADPE), have been reported to induce osteoblast differentiation both *in vitro* and *in vivo* [14]. We previously demonstrated that the CA derivative N-(4-methoxyphen) methyl caffeamide (MPMCA) has greater hepatoprotection than CA under conditions of oxidative stress. MPMCA is purified and obtained from CA through several steps of chromatography on silica gel. Its antioxidant effect is demonstrated by the reduction in thiobarbituric acid reactive substrates, a biomarker of lipid peroxidation, in HepG2 cells [17]. Here, we demonstrate that MPMCA suppresses osteoclast formation and facilitates mature osteoclast apoptosis, suggesting that MPMCA is a potential candidate for treating bone loss disorders.

## MATERIALS AND METHODS

### Materials

**Figure 1:**
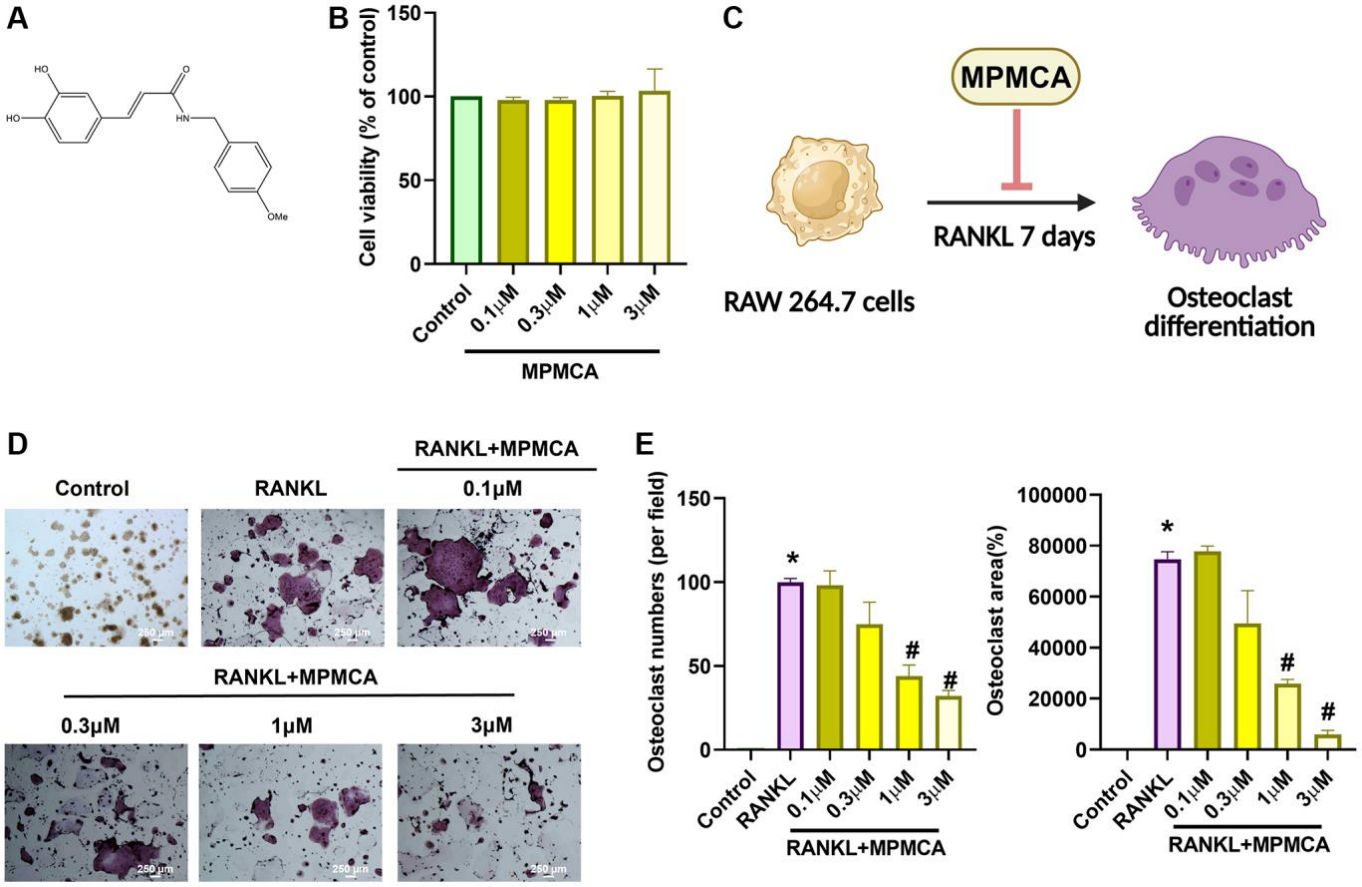
**MPMCA effectively suppresses the formation of osteoclasts induced by RANKL.** (**A**) Diagram illustrating the structure of MPMCA. (**B**) MTT assays were performed to evaluate the viability of RAW 264.7 cells after being treated with MPMCA for 24 hours (*n* = 3). (**C**) Schematic illustrating the inhibitory effect of MPMCA on the process of osteoclast development. (**D**, **E**) TRAP staining was conducted to quantify the number of osteoclasts in RAW 264.7 cells after 7 days of treatment with RANKL and MPMCA (*n* = 3). ^*^p < 0.05 vs. control group. ^#^*p* < 0.05 vs. RANKL-treated group.

MPMCA was synthesized by Dr. Yueh-Hsiung Kuo at China Medical University, Taiwan, following the procedure outlined in a previous report (Figure 1A) [17]. Human recombinant RANKL was purchased from PeproTech, located in Rocky Hill, NJ, USA.

### Cell cultures

RAW 264.7 and MC3T3-E1 cell lines were acquired from the Bioresource Collection and Research Center located in Hsinchu, Taiwan. The cells were cultured in Dulbecco’s Modified Eagle Medium (DMEM) commercial medium obtained from Gibco, Waltham, MA, USA, supplemented with 10% FBS (Gibco, Waltham, MA, USA), as well as penicillin and streptomycin. The cell cultures were maintained at 37°C in a humidified atmosphere containing 5% CO_2_.

### Database analysis

We retrieved a dataset (GSE21639) from the Gene Expression Omnibus (GEO) database, encompassing information pertinent to the genes implicated in the differentiation of RAW 264.7 cells into osteoclasts. This dataset will be instrumental in assessing the expression levels of osteoclast markers during the osteoclast differentiation process [18]. The analysis aimed to identify genes that exhibited statistically significant changes, with a *p*-value less than 0.05 and a fold change ranging from −2 to 2. The KEGG database was subsequently queried using the genes as inputs to elucidate the fundamental pathways associated with osteoclast differentiation. Subsequently, the most upregulated genes were chosen to generate a heatmap, which facilitated the visualization of their expression patterns.

### MTT assay

Cell viability was performed using an MTT test, as described in our previous publications [19, 20]. Concisely, cells were distributed in 96-well plates with a density of 5000 cells per well. After exposing the cultures to different doses of MPMCA for 24 hours, they were washed with PBS before adding a 0.5 mg/mL MTT solution. Afterward, the cells were placed in an incubator at a temperature of 37°C for a duration of 1 h in order to evaluate the vitality of the cells.

### Quantitative real-time PCR

The StepOnePlus sequence detection system was used to conduct quantitative reverse transcription-polymerase chain reaction (qRT-PCR) experiments, following a defined procedure [18, 20–22]. RNA was isolated from RAW 264.7 cells. The reverse transcription of total RNA to complementary DNA (100 ng) was performed using a M−MLV RT kit from Invitrogen, (Thermo Fisher Scientific, Waltham, MA, USA). The StepOnePlus^™^ Real-Time PCR System (Applied Biosystems, Foster City, CA, USA) was employed to amplify the converted cDNA with primers (primers utilized in the qPCR assays are listed in Supplementary Table 1) [23].

### Osteoclast differentiation

The RAW 264.7 cells were cultivated in 24-well plates with a density of 2000 cells per well. Afterward, the cells were subjected to RANKL (50 ng/mL) with MPMCA. Based on our previous investigation, we classified cells with TRAP-positive staining and at least three nuclei as mature osteoclasts after incubating them for 7 days using the TRAP kit from Sigma-Aldrich (St. Louis, MO, USA) [19].

### Western blot analysis

Cell lysates were obtained by employing RIPA buffer. The protein concentrations in each cell lysate were measured using the Pierce^™^ BCA Protein Assay Kit (#23225; Thermo Scientific, Waltham, MA, USA). The electrophoresis and transfer techniques adhered to the established protocols outlined in our previous studies [20, 21, 24]. The membranes were examined using the following antibodies: p-p38, p-ERK, p-JNK, p-p65, ERK, JNK, p38, p65, anti-mouse, and anti-rabbit secondary antibody (Santa Cruz Biotechnology, Inc., Santa Cruz, CA, USA) (antibodies used in the Western blot assays are listed in Supplementary Table 2). The iBright^™^ Imaging System (#CL1500, Invitrogen, Thermo Fisher Scientific, Waltham, MA, USA) was applied to detect luminescence signals.

### Immunofluorescence

The cells were immobilized in a solution containing 3.7% paraformaldehyde for a duration of 30 minutes. Subsequently, they were made permeable by treating them with a 0.1% Triton^™^ X-100 solution for 10 minutes. Finally, the cells were obstructed by incubating them in a 1% BSA solution for 30 minutes. Afterward, the cells were incubated overnight with Cleaved Caspase-3 antibody (Cell Signaling Technology, Danvers, MA, USA), rinsed with PBS, and then treated with Alexa Fluor 488 (Thermo Fisher Scientific, Hemel Hempstead, UK) for a duration of 1 hour. The F-actin rings were labeled using Rhodamine Phalloidin solution (Thermo Fisher Scientific, Waltham, MA, USA) for a duration of 1 hour. The nuclei were labeled using 4,6-diamidino-2-phenylindole (DAPI) for a duration of 15 minutes. The examination of immunofluorescence-stained cells was conducted using a fluorescent microscope (ImageXpress^®^ Pico Automated Cell Imaging System, Invitrogen, Thermo Fisher Scientific, Waltham, MA, USA).

### Statistics

The values are reported as means with their corresponding standard deviations. The statistical significance of the differences between the experimental groups and the control group was assessed using the student’s *t*-test. The data analysis and chart layouts were performed using GraphPad Prism 8.2.1 software from San Diego, CA, USA. Between-group differences were deemed statistically significant if the *p*-value was less than 0.05.

## RESULTS

### MPMCA suppresses osteoclast differentiation

**Figure 2:**
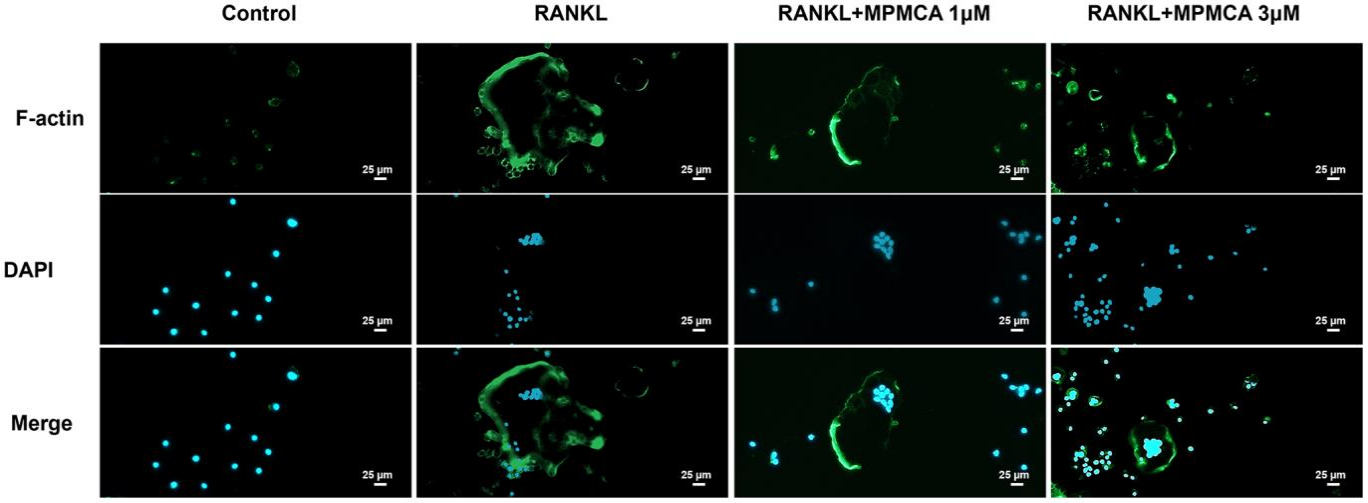
**MPMCA inhibits RANKL-induced F-actin expression.** The RAW 264.7 cells were exposed to RANKL and MPMCA at doses of 1 μM and 3 μM respectively, for a period of 7 days (*n* = 3). The F-actin is represented by green, whilst the nuclei are stained with DAPI and presented in blue.

CA has been shown to effectively inhibit osteoclast development, as established by prior *in vitro* studies [25]. The objective of our investigation was to define the suppressive functions of MPMCA on the process of osteoclast differentiation. Upon initial analysis, the vitality of osteoclast precursor cells was examined after 24 hours. The results showed that there were no notable variations between the groups that were applied with different concentrations of MPMCA (0.1, 0.3, 1, or 3 μM) (Figure 1B). We subsequently examined the prolonged effects of RANKL treatment alone or in conjunction with MPMCA (at concentrations of 0.1, 0.3, 1, or 3 μM) on the osteoclast differentiation derived from RAW 264.7 cells for a period of 7 days (Figure 1C). TRAP staining conducted at the conclusion of this time frame revealed that the inhibition of osteoclast development by MPMCA + RANKL was dependent on the dosage administered. In addition, the combination of MPMCA and RANKL resulted in a reduction in the number of osteoclasts and the area they occupy, with the reduction being dependent on the dosage used (Figure 1D, 1E). Our findings also demonstrate that MPMCA reduces the synthesis of F-actin, which is responsible for the attachment of mature osteoclasts to bone via the podosome belt. This is evident from the observed decrease in RANKL-induced F-actin formation (Figure 2).

**Figure 3:**
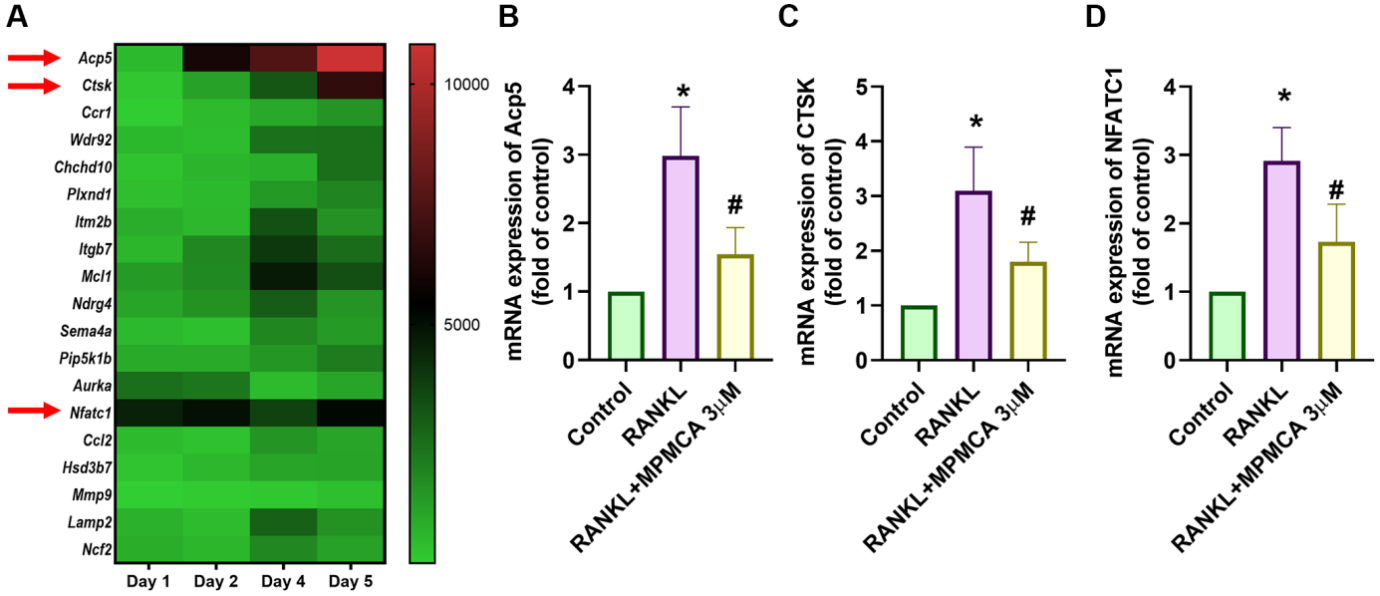
**MPMCA inhibits the expression of osteoclast markers.** (**A**) The GEO database (GSE21639) stores data showcasing the expression levels of osteoclast markers throughout the process of differentiating RAW 264.7 cells into osteoclasts by RANKL treatment. (**B**–**D**) qRT-PCR was used to assess the mRNA expression of osteoclast markers in RAW 264.7 cells. RAW 264.7 cells were exposed to RANKL and MPMCA for a period of 5 days (*n* = 3). ^*^p < 0.05 vs. control group. ^#^*p* < 0.05 vs. RANKL-treated group.

In order to determine the significance of RANKL and gene expression in the process of osteoclastogenesis in RAW 264.7 cells, we investigated the GSE21639 dataset obtained from the GEO database. The dataset involved the application of RANKL to RAW 264.7 cells for a duration of 5 days. Subsequently, the RNA-sequencing data obtained were analyzed (Figure 3A). Our research showed a notable increase in the expression of genes linked with osteoclast development, such as TRAP, CTSK, and NFATC1. MPMCA inhibits RANKL-induced TRAP, CTSK, and NFATC1 expression (Figure 3B–3D). To summarize, our results collectively demonstrate that MPMCA hinders the process of osteoclast differentiation mediated by RANKL.

### MPMCA promotes apoptosis in mature osteoclasts

**Figure 4:**
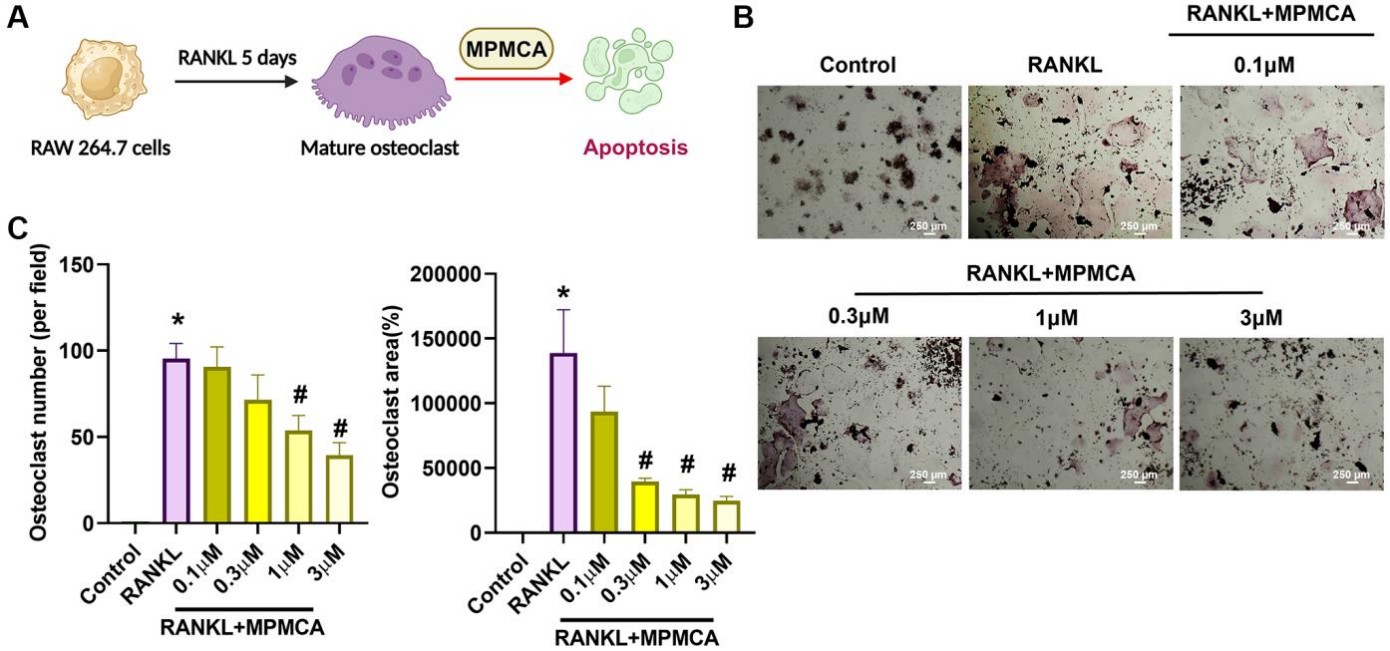
**MPMCA induces apoptosis in mature osteoclasts.** (**A**) The RAW 264.7 cells were cultured with RANKL for 5 days, followed by treatment with RANKL and MPMCA for an additional 2 days. (**B**) TRAP staining was performed on RAW 264.7 cells. (**C**) Quantitative results derived from TRAP staining in RAW 264.7 cells (*n* = 3). ^*^p < 0.05 vs. control group. ^#^*p* < 0.05 vs. RANKL-treated group.

**Figure 5:**
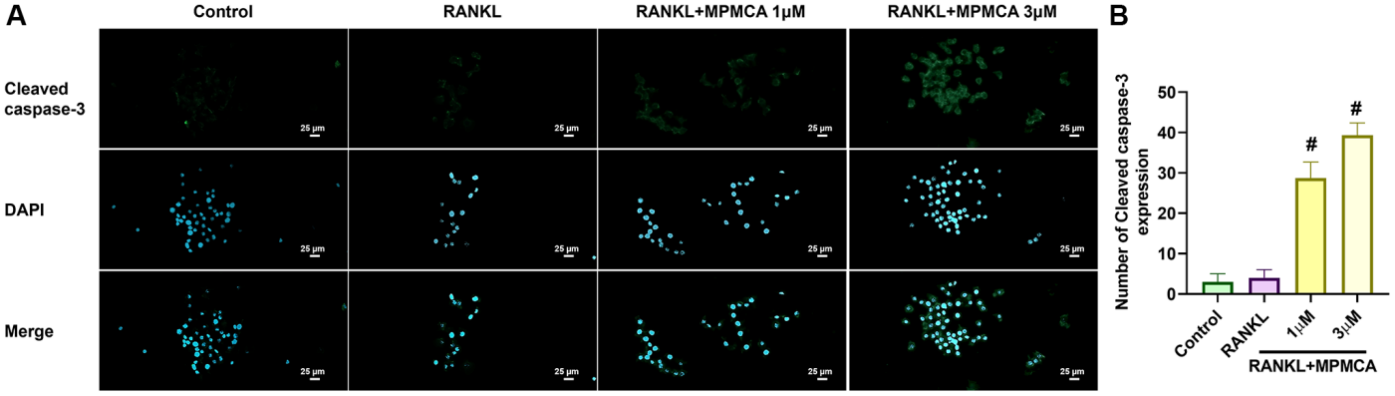
**MPMCA enhances the level of cleaved caspase-3 in mature osteoclasts.** (**A**) Immunofluorescence staining was performed on RAW 264.7 cells to detect cleaved caspase-3. The cells were initially stimulated with RANKL for 5 days, and then treated with RANKL and MPMCA for an additional 2 days. Cleaved caspase-3 is indicated by green, while the staining for nuclei using DAPI is shown in blue. (**B**) The enumeration of cells that had positive staining was conducted (*n* = 3). ^*^p < 0.05 vs. control group. ^#^*p* < 0.05 vs. RANKL-treated group.

We next examined the effects of MPMCA on mature osteoclasts. The RAW 264.7 cells were first applied to 50 ng/ml RANKL for a period of 5 days to stimulate the mature osteoclasts. Subsequently, the cells were then exposed to various concentrations of MPMCA (0.1, 0.3, 1, or 3 μM) for a duration of 48 hours (Figure 4A). TRAP staining demonstrated a notable decrease in the lifespan of mature osteoclast cell differentiation caused by MPMCA treatment (Figure 4B). The treatment with MPMCA resulted in an inhibition in both the number and size of osteoclasts (Figure 4C). Further confirmation of apoptosis in mature osteoclasts produced by MPMCA was achieved through further labeling with Cleaved caspase-3 and DAPI (Figure 5A, 5B). Therefore, our results indicate that MPMCA induces cell apoptosis in mature osteoclasts.

### MPMCA regulates MAPKs and NF-κB signaling pathways in RAW 264.7 cells

**Figure 6:**
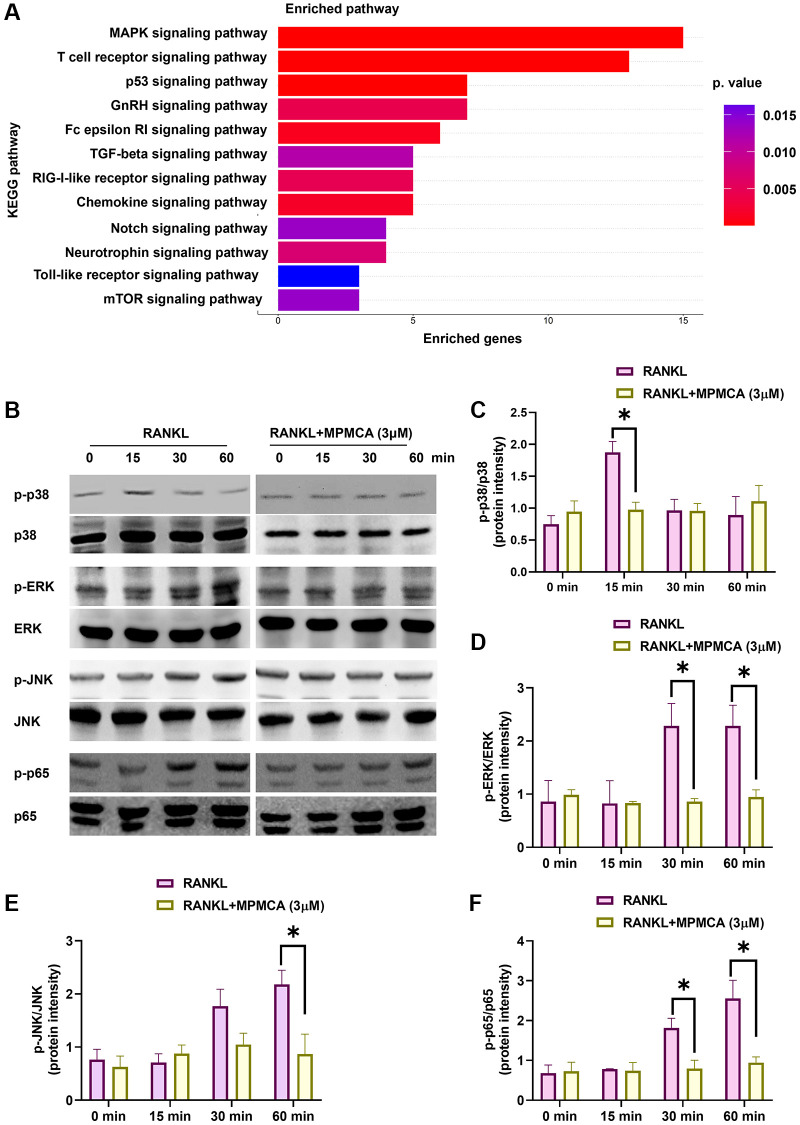
**MPMCA hinders the activation of MAPKs and NF-κB.** (**A**) KEGG pathway database map illustrating the significantly upregulated pathways in the GSE21639 database. (**B**–**F**) Western blot analysis reveals the phosphorylation of MAPKs and p65 in RAW 264.7 cells following treatment with RANKL and MPMCA at specific time intervals (*n* = 3). ^*^p < 0.05 vs. RANKL-treated group.

Prior research has emphasized the participation of several RANKL-mediated signaling pathways in the functioning of osteoclasts [25–27]. Upon analyzing the RNA-sequencing results obtained from the GEO dataset GSE21639, we detected a significant upregulation in the expression of many pathways, such as MAPKs (p38, JNK, ERK) and NF-κB, after administering RANKL treatment (Figure 6A). Given these discoveries, our study sought to examine the impact of MPMCA treatment on MAPKs and NF-κB signaling. Western blot analyses were conducted on RAW 264.7 cells that were treated with MPMCA at concentrations of 1 μM and 3 μM. The results indicated that RANKL-induced phosphorylation of MAPKs and p65 decreased in a concentration-dependent manner after MPMCA treatment (Figure 6B–6F). The findings indicate that MPMCA hinders the activity of osteoclasts by regulating the MAPKs and NF-κB signaling pathways.

### MPMCA does not stimulate the expression of osteoblast differentiation markers

**Figure 7:**
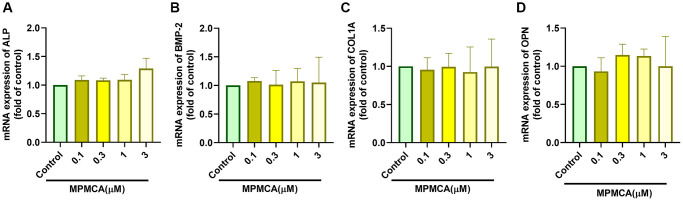
**MPMCA does not induce the upregulation of osteogenic markers in osteoblasts.** (**A**–**D**) MC3T3-E1 cells were subjected to predetermined concentrations of MPMCA for a period of 24 hours. RT-qPCR test was performed to examine the mRNA levels of osteogenic markers (*n* = 3). ^*^p < 0.05 vs. control group.

Prior research has indicated that CA derivatives have the ability to promote the development of osteoblasts and increase bone mass [25, 28]. In order to examine the influence of MPMCA on the process of osteoblast differentiation, we evaluated the levels of expression of certain markers associated with osteoblast differentiation, such as ALP, BMP-2, COL1A, and OPN. Nevertheless, our findings demonstrated that MPMCA had no substantial effect on the expression of these osteoblast differentiation markers (Figure 7). Based on our data, it may be inferred that MPMCA does not stimulate osteoblast differentiation.

## DISCUSSION

CA has been acknowledged for its capacity to regulate bone remodeling by inhibiting the formation of osteoclasts, the process of bone resorption, and cell apoptosis of osteoclasts. As a result, it demonstrates therapeutic properties in the treatment of bone diseases [16]. The aim of this study is to investigate the impact of MPMCA, a modified variant of CA, on the process of RANKL-induced osteoclastogenesis in RAW 264.7 cells. The results of our investigation demonstrate that MPMCA has the dual effect of decreasing the differentiation of osteoclasts and causing cell apoptosis in mature osteoclasts. Additionally, it simultaneously inhibits the activation of ERK, p38, and JNK as well as NF-κB, which are pathways enhanced by RANKL. These findings suggest that MPMCA has potential as a strong option for the advancement of treatments that prevent bone loss.

The maintenance of skeletal homeostasis depends on the dynamic equilibrium between osteoblasts and osteoclasts, which contribute to the ongoing processes of bone production and resorption [29, 30]. Osteoporosis, which is defined by reduced bone density, weakened bone structure, and impaired microarchitecture, increases the likelihood of fractures [31]. Osteoclasts, which are stimulated by RANKL, have a crucial function in both normal and abnormal bone breakdown [32, 33]. We employed murine macrophages (RAW 264.7 cells) in our research to produce osteoclasts via RANKL activation in a laboratory setting. Our objective was to evaluate the function of MPMCA on the formation of osteoclasts. Our findings indicate that MPMCA has the novel effect of decreasing the development of osteoclasts and triggering programmed cell death in mature osteoclasts. This may be due to its capacity to hinder the differentiation of osteoclast precursors and enhance cell apoptosis in mature osteoclasts.

MAPKs, an essential type of signaling pathway, have a central function in the generation and differentiation of osteoclasts. Their activation is triggered by cytokines like RANKL [26, 27]. Prior research has shown that chemicals derived from caffeic acid hinder the activation of MAPKs pathways and obstruct osteoclastogenesis by decreasing the expression of osteoclast differentiation markers [34–36]. Our examination of data from the GEO dataset has substantiated the correlation between MAPKs mechanism, specifically the MAPK signaling pathway. The application of RANKL to RAW 264.7 cells resulted in the enhancement of ERK, p38 and JNK phosphorylation. In contrast, when the cells were treated with MPMCA, it was observed that the effects were reversed. This indicates that MPMCA is involved in the activation of the ERK, p38 and JNK signaling cascades, which in turn leads to a decrease in the formation of osteoclasts induced by RANKL, as well as a decrease in the expression of mRNA for osteoclast markers.

The investigation into the use of TCM as a possible remedy for osteoporosis has attracted considerable interest. Several TCM treatments have shown dual benefits, displaying both anabolic qualities that stimulate bone development and anticatabolic actions that prevent bone resorption [37–39]. Prior studies have emphasized the ability of CA derivatives to suppress the activity of genes that play a role in the formation of osteoclasts [34, 36]. Consistent with these discoveries, our data demonstrate that MPMCA greatly boosts the expression of genes linked to the formation of osteoclasts, such as Acp5, Cathepsin K, and NFATC1. The use of CA led to an increase in the expression of genes related to osteoblasts, specifically BMP-2, BMP-7, TGF-β1, RUNX-2 and ALP [40]. However, our analysis showed that the use of MPMCA did not cause a notable rise in the levels of osteogenic markers, including ALP, BMP-2, COL1A, and OPN in osteoblasts. Therefore, MPMCA exhibits different effects in osteoblasts compared with CA. The most critical structure remains obscure due to the structural differences. Nevertheless, we have recognized the issue and suggest that additional research be conducted. Furthermore, MPMCA has the potential to be used in the treatment of bone resorptive disorders, including osteoporosis and arthritis, and whether it can be applied to clinical treatments needs further examination.

The advantageous effects of CA and its derivatives on bone health have been demonstrated in previous studies [41, 42]. Folwarczna et al. reported that the mechanical properties of bone were enhanced by the administration of CA (5 and 50 mg/kg via stomach tube for 4 weeks) in ovariectomized (OVX) rats. This was achieved by increasing the width of the trabecular metaphysis and decreasing transverse development in the endosteal region of the femur [41]. The most extensively investigated derivative of caffeic acid in animal studies, CAPE, has demonstrated beneficial effects on new bone formation and regeneration subsequent to systemic administration [28, 43–45]. Duan et al. found that a lower dose and frequency of CAPE injection (0.5 mg/kg twice a week, intraperitoneally for 4 weeks) increased bone volume and trabecular number by reducing osteoclast formation, as evidenced by a decreased osteoclast number per bone perimeter, in OVX rats [42]. The difficult synthesis of MPMCA in this study led to inadequate quantities, which hindered the possibility of conducting an *in vivo* investigation to determine whether MPMCA exhibits an anti-resorption effect in animal models that need further investigation.

**Figure 8:**
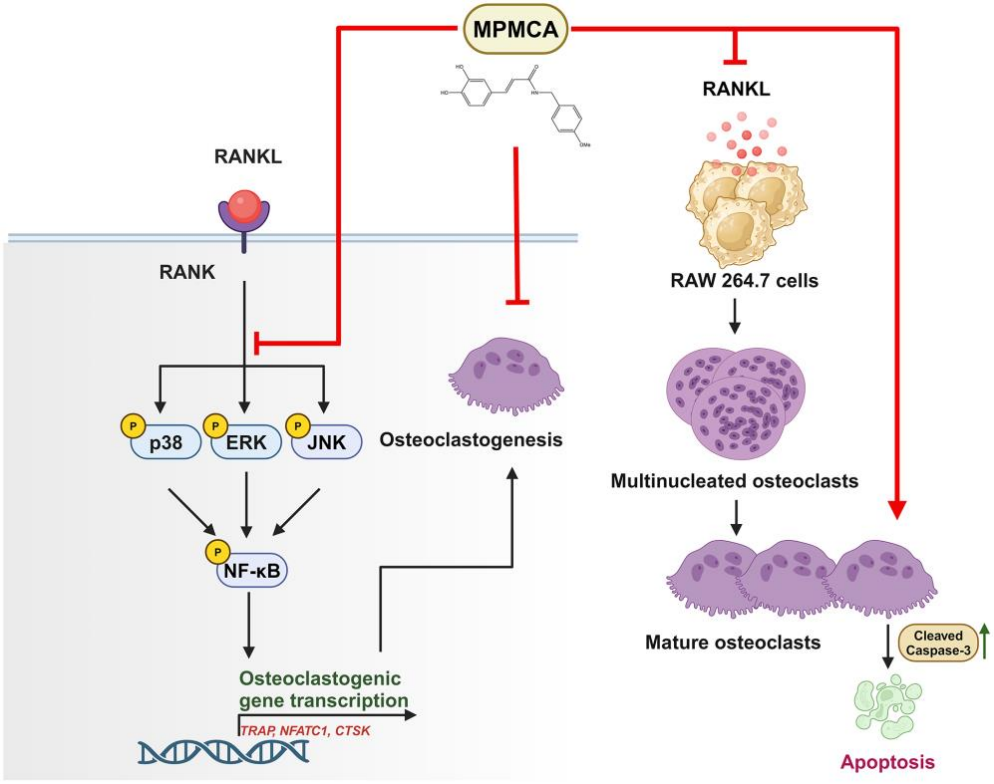
**The graphic illustrates the signaling pathways impacted by MPMCA, leading to the suppression of osteoclast activity.** MPMCA hinders the transformation of macrophages into osteoclasts and intensifies the cell apoptosis of mature osteoclasts. MPMCA facilitates the inhibition of MAPKs (ERK, p38 and JNK) and NF-κB signaling pathways.

The study findings indicate that MPMCA hinders the formation of osteoclasts from macrophages, induces apoptosis in mature osteoclasts, and inhibits the MAPK and NF-κB signaling pathways stimulated by RANKL. The findings indicate that MPMCA holds promise as a viable choice for developing novel therapies aimed at mitigating bone loss (Figure 8).

## Supplementary Materials

Supplementary Tables
