# Supplementary material for: Caffeic acid derivative MPMCA suppresses osteoclastogenesis and facilitates osteoclast apoptosis: implications for the treatment of bone loss disorders
Source: Aging (Albany NY). 2024 Aug 26;16(16):11926–38. doi: 10.18632/aging.206067 (PMC11386915; doi:10.18632/aging.206067)
Supplement: Supplementary Tables [file aging-16-206067-s001.pdf]

## SUPPLEMENTARY TABLES

**Supplementary Table 1. Primers that were used in this study.**

| Gene | Forward | Reverse |
|---|---|---|
| GAPDH | TGTGTCCGTCGTGGATCTGA | TTGCTGTTGAAGTCGCAGGAG |
| Acp5 | ATGGGCGCTGACTTCATCAT | GGTCTCCTGGAACCTCTTGT |
| CTSK | AGTAGCCACGCTTCCTATCC | CCATGGGTAGCAGCAGAAAC |
| NFATC1 | GACCCGGAGTTCGACTTCG | TGACACTAGGGGACACATAACTG |
| ALP | TAGCGGTTACTGTAGACACCC | CACCCTCAAGAGCCTGAGTC |
| BMP-2 | TGGAAGTGGCCCATTTAGAG | TGACGCTTTTCTCGTTTGTG |
| COL1A | CACCCTCAAGAGCCTGAGTC | TTAGGCGCAGGAAGGTCAGC |
| OPN | GGGACATCGACTGTAGGGACG | ATCTTTCTGCTCACTCTGCT |

**Supplementary Table 2. Antibodies that were used in this study.**

| Protein | Dilution | Catalog No. | Source |
|---|---|---|---|
| p-p38 | 1:3000 | SC-166182 | Santa Cruz Biotechnology, Dallas, TX, USA |
| p38 | 1:3000 | SC-7972 | Santa Cruz Biotechnology, Dallas, TX, USA |
| p-ERK | 1:3000 | SC-7383 | Santa Cruz Biotechnology, Dallas, TX, USA |
| ERK | 1:3000 | SC-1647 | Santa Cruz Biotechnology, Dallas, TX, USA |
| p-JNK | 1:3000 | SC-6254 | Santa Cruz Biotechnology, Dallas, TX, USA |
| JNK | 1:3000 | SC-7345 | Santa Cruz Biotechnology, Dallas, TX, USA |
| p-p65 | 1:3000 | SC-101752 | Santa Cruz Biotechnology, Dallas, TX, USA |
| p65 | 1:3000 | SC-8008 | Santa Cruz Biotechnology, Dallas, TX, USA |
